# PGPR-mediated enhancement of growth, phytochemical diversity, and metabolites in black turmeric (*Curcuma caesia* Roxb)

**DOI:** 10.3389/fmicb.2026.1787961

**Published:** 2026-03-13

**Authors:** Ni Luh Suriani, Ting Seng Ho, Nadiah S. Alzahrani, Dewa Ngurah Suprapta, I. Nyoman Suarsana, Ni Made Delly Resiani, Riyaz Sayyed

**Affiliations:** 1 Program Study Magister of Sustainable Finance and Development, Post Graduate, Biology Study Program, Faculty of Mathematics and Natural Sciences, Udayana University, Bali, Indonesia; 2 Back2Nature Regenerative Farm, Kuala Pilah, Malaysia; 3 College of Science, Al Baha University, Al Baha, Saudi Arabia; 4 Biopesticide Laboratory, Agriculture Faculty, Udayana University, Bali, Indonesia; 5 Biochemistry Laboratory, Faculty of Veterinary Medicine, Udayana University, Bali, Indonesia; 6 National Research and Innovation Agency, Jakarta, Indonesia; 7 Department of Microbiology, PSGVP Mandal's S I Patil Arts, G B Patel Science and STSK Sangh Commerce College, Shahada, India

**Keywords:** *Bacillus subtilis*, bioactive compounds, GC–MS analysis, medicinal plants, *Paenibacillus polymyxa*, phytochemical profiling, root colonization, secondary metabolites

## Abstract

**Introduction:**

Medicinal plants are an essential source of bioactive compounds with therapeutic potential, yet their cultivation frequently depends on chemical fertilizers and pesticides that may compromise environmental and human health. Plant growth–promoting rhizobacteria (PGPR) represent a sustainable alternative for enhancing plant productivity and phytochemical quality through beneficial plant–microbe interactions. This study evaluated the effects of rhizobacterial inoculation on growth performance, phytochemical composition, and secondary metabolite production in black turmeric (*Curcuma caesia*), a medicinal plant recognized for its anticancer properties.

**Methods:**

A greenhouse experiment was conducted using a completely randomized design with five treatments: inoculation with *Bacillus subtilis*, *Paenibacillus polymyxa*, a bacterial consortium (*B. subtilis* + *P. polymyxa*), NPK fertilizer, and an uninoculated control. Rhizobacteria were applied at a concentration of 2%. Each treatment consisted of five replicates, with three plants per replicate (total = 75 plants). Plant growth parameters, dry biomass, phytochemical profiles, GC–MS analysis, and bacterial colony–based secondary metabolite analysis were assessed.

**Results:**

Rhizobacterial treatments significantly enhanced plant growth, dry weight, and phytochemical content compared to the control and NPK treatment. *B. subtilis* inoculation resulted in the highest growth performance and the greatest diversity of phytochemical compounds detected by GC–MS. The consortium treatment exhibited the highest abundance and area of secondary metabolite compounds, indicating synergistic interactions between the two bacterial strains. Both *B. subtilis* and *P. polymyxa* demonstrated traits associated with PGPR activity, including indole-3-acetic acid production, nitrogen fixation, phosphate solubilization, root colonization, and induction of systemic resistance, contributing to improved nutrient uptake and suppression of wilt disease.

**Discussion:**

These findings highlight the role of PGPR-mediated mechanisms in enhancing secondary metabolite biosynthesis and medicinal quality in black turmeric. The use of rhizobacterial inoculants, particularly *B. subtilis* and its consortium with *P. polymyxa*, offers a promising strategy for sustainable cultivation of medicinal plants and supports the integration of microbial biotechnology in sustainable pharmacological production.

## Introduction

Advances in modern medicine have progressed rapidly, particularly through the development of chemical-based treatments due to their rapid and practical therapeutic effects. However, prolonged or excessive use of synthetic drugs is often associated with adverse side effects, prompting increased global interest in alternative and complementary medicines, especially herbal-based therapies. Herbal medicines are generally regarded as safer, with fewer side effects, owing to their natural origin and bioactive phytochemical constituents. Plants contain a wide range of secondary metabolites, including alkaloids, flavonoids, tannins, saponins, and antioxidants, which exhibit significant medicinal and preventive properties against various diseases (Latif and Nawaz, 2025; Sun et al., 2024).

The Earth’s biodiversity offers an abundant reservoir of medicinal plants that have been traditionally utilized for centuries (Cruciani et al., 2026). Classical medical systems such as Ayurveda (Wallace, 2020) and traditional Chinese medicine (Kang et al., 2025) extensively employ plant-based remedies for disease prevention and treatment. Numerous tropical plants, including *Moringa oleifera*, *Ocimum basilicum*, *Carica papaya*, ginger, galangal, turmeric (yellow, white, and black), and several others, possess high potential for development as medicinal plants (Latif and Nawaz, 2025).

Black turmeric (*Curcuma caesia*) has gained increasing attention due to its rich phytochemical profile and broad pharmacological activities. Previous studies have reported that black turmeric exhibits anti-inflammatory, anticancer, antibacterial, antioxidant, and anti-eczema properties, and is effective in alleviating skin irritation and itching (Zoi et al., 2021; Razak et al., 2024). Given its medicinal significance, ensuring high-quality cultivation practices is essential to maximize its therapeutic value.

The continued use of chemical fertilizers and pesticides in medicinal plant cultivation can compromise product quality by introducing harmful chemical residues that pose health risks (Ádám et al., 2024). Consequently, researchers worldwide are increasingly focusing on sustainable agricultural practices, including the use of rhizobacteria, organic fertilizers and biopesticides, which are environmentally friendly, biodegradable, and target-specific in their action (Khairnar et al., 2025; Suriani et al., 2021). Among these approaches, the application of plant growth–promoting rhizobacteria (PGPR) has received considerable attention due to their safety and multifunctional benefits, such as phytohormone production, biological pest control, and nutrient mobilization. This biotechnology aligns strongly with the principles of sustainable agriculture and the United Nations Sustainable Development Goals (SDGs; Espinosa-Palomeque et al., 2025; de Andra et al., 2023).

Several rhizobacterial strains, including *Brevibacillus agri*, *Bacillus velezensis*, *Paenibacillus polymyxa*, and *Pseudomonas monteilii*, have been shown to significantly enhance plant growth and phytochemical content in medicinal plants such as Javanese ginseng (Suriani et al., 2024). *Paenibacillus polymyxa* is known for its antagonistic activity against pathogens, nitrogen-fixing ability, and plant growth–promoting effects (Simarmata et al., 2025). Similarly, *B. subtilis* functions as an effective PGPR, enhancing plant growth and physiological performance (Sagar et al., 2024; Ilyas et al., 2022; Nithyapriya et al., 2021)*. B. subtilis* is a PGPR that functions as a biostimulant, biofertilizer, and biopesticide. *B. subtilis* is used as a biocontrol agent because it can suppress plant diseases. It produces antibiotics, hydrolytic enzymes, and volatile organic compounds that help in suppressing fungal pathogens (Adjei et al., 2025). The use of PGPR as biocontrol agent offers several advantages over their chemical counterparts (Al Raish et al., 2025).

Given the medicinal importance of black turmeric and the growing demand for high-quality herbal raw materials free from chemical residues, it is essential to investigate sustainable cultivation strategies. Therefore, this study aims to evaluate the influence of rhizobacteria on the growth, yield, and phytochemical content of black turmeric (*C. caesia*) as a potential approach to improving the quality of herbal medicinal products.

## Materials and methods

### Source of microbial cultures

The rhizobacterial strains *Paenibacillus polymyxa* (F1; GenBank Accession No. OR244033) and *Bacillus subtilis* (F2; GenBank Accession No. OR225824) previously isolated by researchers of Back2Nature Laboratory, Kuala Pilah, Negeri Sembilan, Malaysia were used in the present study.

### Time and location of the study

The experiment was conducted between January 2025 and December 2025 at the Biopesticide Laboratory, Udayana University, Denpasar, Bali, Indonesia, and at Dewandaru Flora Organic Farm, Tabanan, Bali, Indonesia. The Back2Nature Laboratory, the source of microbial cultures, is located in Kuala Pilah, Malaysia (2°44′20″N, 102°14′56″E), which experiences an equatorial climate (Köppen–Geiger classification: Af), with average air temperatures ranging from 25 to 28 °C (Suriani et al., 2024).

### Experimental design

The agricultural experiment followed a randomized block design comprising five treatments with five replicates each, resulting in a total of 25 experimental units. Each unit consisted of three plant clumps, yielding 75 clumps overall.

The treatments were defined as follows:

- F0: Uninoculated control (native soil only).
- F1: *P. polymyxa* (2%).
- F2: *B. subtilis* (2%).
- F3: Consortium of *P. polymyxa* (2%) + *B. subtilis* (2%).
- F4: NPK fertilizer (16–16–16; 2.5 g plant^−1^).

Treatments were applied following protocols described by Suriani et al. (2020).

### Screening of plant growth–promoting traits

#### Indole-3-acetic acid production

Bacterial isolates were cultured in tryptic soy broth and incubated for 48 h at 28 °C in the dark. Following incubation, 1 mL of Salkowski’s reagent was added to each culture. Development of a pink coloration indicated IAA production, which was quantified spectrophotometrically at 520 nm (Guardado-Fierros and Tuesta-Popolizio, 2024).

#### Nitrogen fixation

Nitrogen fixation ability was assessed by culturing bacterial strains in nitrogen-free bromothymol blue malate medium at 28 °C for 48 h. The formation of yellow-colored colonies indicated nitrogen-fixing activity (Shi et al., 2023).

#### Phosphate solubilization

Phosphate solubilization was evaluated by inoculating bacterial cultures onto Pikovskaya’s agar and incubating them at 28 °C for 48 h. The presence of clear halo zones surrounding bacterial colonies indicated phosphate solubilization capacity (Güler, 2024).

### Production of rhizobacterial inoculum

Bacterial cultures were propagated on nutrient agar (NA) medium. Five loopfuls of each culture were inoculated into 1 L of nutrient broth and incubated at 30 °C for 72 h. Cells were harvested by centrifugation at 4,000 rpm for 10 min and resuspended in sterile 0.9% NaCl solution. Cell density was adjusted to 1.5 × 10^8^ CFU mL^−1^, corresponding to a 0.5 McFarland standard (Suriani et al., 2024; Wiegand et al., 2024).

### Greenhouse experiment

#### Preparation of planting medium

Black turmeric (*Curcuma caesia*) tubers were sourced from the Back2Nature Regenerative Farm, Kuala Pilah, Malaysia. Plants were cultivated in 30 cm diameter polybags filled with a planting medium composed of soil, rice husk biochar, and compost (2:1:1, v/v/v). The compost was produced in-house using cow manure, goat manure, and agricultural residues (Suriani et al., 2019).

#### Seed preparation and planting

For each treatment, 1 kg of black turmeric tubers was soaked for 30 min prior to sowing. Treatments included soaking in water (F0 and F4), *P. polymyxa* (F1), *B. subtilis* (F2), or a bacterial consortium (F3; Maulina et al., 2022). Tubers were pre-grown for 2 months, after which uniform seedlings (~20 cm height) were selected and transplanted vertically at a depth of approximately 10 cm (Suriani et al., 2019).

#### Application of treatments and crop management

Rhizobacterial inocula were applied weekly from planting until plants reached 3 months of age. Treatments F1, F2, and F3 received 100 mL of 2% bacterial suspension (1.5 × 10^8^ CFU mL^−1^) per plant. Treatment F4 received NPK fertilizer (2.5 g dissolved in 100 mL water per plant) every 3 months. Control plants (F0) received water only.

All plants received routine maintenance, including compost application (300 g plant^−1^ every 3 months), weeding, and regulated irrigation once per week to induce mild stress and promote uniform growth (Farouk et al., 2024; Vafa et al., 2021).

### Harvesting and growth assessment

Plants were harvested 8 months after planting. Tubers were cleaned and air-dried to remove surface moisture. Growth parameters measured included plant height, root length, leaf area, chlorophyll content, fresh weight, and dry weight. Soil samples were collected for N, P, and K analysis. Phytochemical analyses included total phenolics, flavonoids, tannins, alkaloids, antioxidant activity, and antioxidant capacity.

### Disease incidence

Disease incidence was assessed based on symptoms of wilting, premature leaf yellowing, stem rot, and plant death. Disease percentage was calculated using the formula (Kasim, 2024).


$$\text{Disease Percentage}\;(\%)=\frac{\text{Number of Plants Affected}\;\mathrm{by}\;\text{Disease}}{\text{Number of plants}\;\mathrm{all}}x100$$

### Preparation of plant extracts

Air-dried black turmeric tubers were powdered and extracted with ethanol. Extracts were concentrated using a rotary evaporator and used for subsequent phytochemical and antioxidant analyses (Lee et al., 2021).

### Estimation of chlorophyll

Chlorophyll measurement using a SPAD 502 meter, the SPAD 502 meter measures the greenness level of leaves based on red (~650 nm) and infrared (~940 nm) light transmittance. The value of Soil and Plant Analysis Development (SPAD) is directly correlated with the chlorophyll content and nitrogen status of the leaves (Shah et al., 2017).

### Phytochemical and antioxidant analyses

Total phenolic content, flavonoids, tannins, alkaloids, and antioxidant activity using 1-1-diphenyl-2-picrylhydrazyl (DPPH) assay were quantified using standard colorimetric and spectrophotometric methods as previously described (Khoddami et al., 2013; Singleton et al., 2022).

### GC–MS analysis

Gas chromatography–mass spectrometry (GC–MS; QP2010SE, Shimadzu, Japan) was performed to identify bioactive compounds in black turmeric extracts. Compound identification was achieved by comparing mass spectra with those in the GC–MS library database (Reshma et al., 2018; Suriani et al., 2020). Analyses were conducted at the Joint Mathematics and Natural Sciences Laboratory, Udayana University, Bali, Indonesia.

### Scanning electron microscopy analysis

Root colonization by rhizobacteria was examined using field-emission scanning electron microscopy (FE-SEM). Treated and untreated root samples were dehydrated, dried, and analyzed under vacuum conditions. Imaging was conducted at an accelerating voltage of 3 kV, while energy-dispersive X-ray (EDX) analysis was performed at 15 kV (Zhang et al., 2020; Suriani et al., 2024). SEM analysis was carried out at Universitas Gadjah Mada (UGM), Indonesia.

### Statistical analysis

All experimental data were analyzed using SPSS software. One-way analysis of variance (ANOVA) was performed, and mean comparisons were conducted using Duncan’s multiple range test at a significance level of *p* ≤ 0.05 (Hosseini et al., 2022; Yan et al., 2024).

## Results

### Plant growth–promoting traits of rhizobacteria

Both *P. polymyxa* and *B. subtilis* strains were selected for their known plant growth-promoting traits, including IAA production, phosphate solubilization, and nitrogen fixation. Colorimetric and qualitative assays confirmed their capacity to synthesize IAA, form nitrogen-fixing colonies, and produce clear halo zones on Pikovskaya’s medium, demonstrating their functional roles in plant-microbe interactions.

### Soil nutrient status after treatments

Soil analysis conducted after harvest revealed significant differences in nutrient content among treatments, highlighting the influence of rhizobacteria on soil health through mechanisms such as nutrient mobilization and mineralization (Table 1). The highest soil nitrogen content (0.67%) was recorded in the NPK treatment, while the *B. subtilis* (F2) treatment showed the highest phosphorus (1,306,452 mg kg^−1^) and potassium (698,334 mg kg^−1^) levels. All rhizobacterial treatments significantly increased N, P, and K levels compared to the control, indicating their role in enhancing soil fertility and microbial-driven nutrient cycling.

**Table 1:** Soil analysis after 8 months of treatment.

Parameters	Treatments				
	F0	F1	F2	F3	F4
Nitrogen (%)	0.37 ± 0.62a	0,45 ± 0.32b	0,46 ± 0.22b	0,57 ± 0.44c	0.67 ± 0.42d
Phosphorus(mg/kg)	1.266,332 ± 0.12a	1.311,122 ± 0.34c	1.326,254 ± 0.041c	1.306,452 ± 0.13b	1.302,352 ± 0.17b
Potassium (mg/kg)	560,721 ± 31a	697,285 ± 15b	698,334 ± 41b	691,935 ± 0.51b	680,721 ± 31b

Values represent the average of triplicates, with ± indicating the standard deviation. Different letters denote significant differences between the values at *p* > 0.05. F0, Control; F1, 2% *B. polymyxa*; F2, 2% *B. subtilis*; F3, 2% consortium of *B. subtilis* and *B. polymyxa*; F4, NPK synthetic.

### Plant growth parameters and chlorophyll content

**Figure 1:**
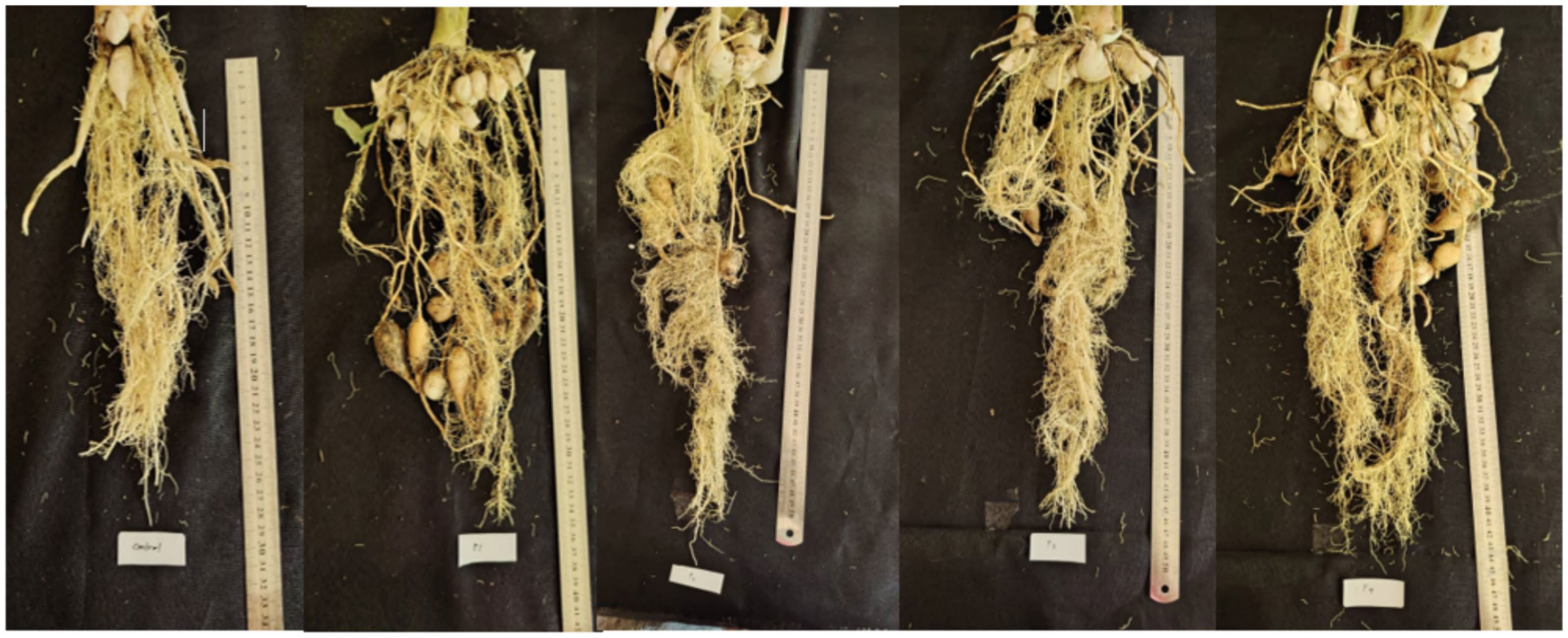
Root of black turmeric following various treatments, from left to right F0, F1, F2, F3, and F4 treated plant roots.

Measurements taken after 4 months aimed to evaluate the effects of treatments on plant growth, including height, leaf area, root length, and chlorophyll content (Figure 1; Table 2). The consortium treatment (F3) resulted in the tallest plants, while the NPK treatment (F4) produced the largest leaf area. Root length was most significant in the *B. subtilis* (F2) treatment, and chlorophyll content peaked in the *P. polymyxa* (F1) treatment. All treatments showed substantial differences from the control, demonstrating their impact on plant development.

**Table 2:** Growth of black turmeric plants after a 4-month planting period.

Treatments	Height (cm)	SPAD Index	Leaf area (cm^2^)	Root length (cm)
F0	128.32 ± 0.22a	51,21 ± 2,52a	715.17 ± 0.33a	30.38 ± 0.52a
F1	150.25 ± 0.24c	58,12 ± 2,76d	855.21 ± 0.15b	36.32 ± 0.12b
F2	142.46 ± 0.13b	57,14 ± 45.7c	860.30 ± 0.12c	54.40 ± 0.71d
F3	166.25 ± 0.21d	54,12 ± 2,2b	832.71 ± 0.18b	47.12 ± 0.32b
F4	150.16 ± 0.12c	57,55 ± 4,74c	865.12 ± 0.23c	46.11 ± 0.34c

Values represent the average of triplicates, with ± indicating the standard deviation. Different letters denote significant differences between the values at *p* > 0.05. F0, Control; F1, 2% *B. polymyxa*; F2, 2% *B. subtilis*; F3, 2% consortium of *B. subtilis* and *B. polymyxa*; F4, NPK synthetic.

### Yield attributes and disease incidence

At harvest (8 months), yield attributes such as fresh and dry rhizome weights varied significantly among treatments (Table 3). The NPK treatment yielded the highest fresh weight, while the *B. subtilis* (F2) treatment produced the highest dry rhizome weight (248.46 g). The NPK treatment also experienced the most significant weight loss during drying (22.3%), emphasizing differences in post-harvest quality. Wilt disease incidence was only observed in the NPK treatment (6.67%), with no symptoms in rhizobacterial or control treatments, indicating potential disease suppression by rhizobacteria.

**Table 3:** Yield of black turmeric plants after an 8-month planting period and % of wilt disease after 6 months of planting.

Treatments	Wet weight (g)	Dry weight (g)	Weight (%)	Wilt disease (%)
F0	1000.12 ± 0.25a	220,03 ± 2,12a	22	%
F1	1120.21 ± 0.54b	246.45 ± 2,16c	22	0%
F2	1123.46 ± 0.13b	248.46 ± 15.7d	22.1	0%
F3	1110.25 ± 0.21b	233.80 ± 1,42b	22	0%
F4	1166.16 ± 0.12b	248.39 ± 4,14d	22.3	6.67%

Values represent the average of triplicates, with ± indicating the standard deviation. Different letters denote significant differences between the values at *p* > 0.05. F0, Control; F1, 2% *B. polymyxa*; F2, 2% *B. subtilis*; F3, 2% consortium of *B. subtilis* and *B. polymyxa*; F4, NPK synthetic.

Only the NPK treatment showed wilt symptoms with 6.67% incidence; all rhizobacterial treatments effectively prevented disease, reassuring the audience of their protective effects and potential for sustainable disease management.

### Phytochemical content and antioxidant activity

Results show that rhizobacterial treatments significantly enhanced phytochemicals and antioxidant activity, highlighting their potential to improve medicinal quality and inspire confidence in sustainable practices (Table 4).

**Table 4:** UV–Vis spectrophotometric analysis of phytochemicals in black turmeric after 8 months of planting.

Results	Unit	Sample				
		F0	F1	F2	F3	F4
Total flavonoid	mg QE/100 mL	68.65 ± 0.84a	76.38 ± 0.72e	71.83 ± 0.28d	70.90 ± 0.63c	69.77 ± 0.81b
Total phenol	mg GAE/100 g	336.81 ± 0.12a	379.40 ± 0.56c	409.52 ± 0.34e	393.47 ± 0.81d	363.65 ± 0.52b
Ic 50%	ppm	4050.52 ± 0.23a	3437.5 ± 0.22d	3,435 ± 0.31e	3687.79 ± 0.61c	4037.09 ± 0.29b
Tannin	mgTAE/100 g	161.519 ± 0.61a	182.27 ± .31e	179.946 ± 0.45d	164.369 ± 0.36b	176.957 ± 0.71c
Antioxidant capacity	ppm GAEAC	773.246 ± 0.24a	899.20 ± 0.62c	954.191 ± 0.39e	908.710 ± 0.76d	872.086 ± 0.23b
Alkaloid	%	0.18 ± 0.11a	0.64 ± 0.30a	1.02 ± 0.36a	0.44 ± 0.42b	0.17 ± 21c

Values represent the average of triplicates, with ± indicating the standard deviation. Different letters denote significant differences between the values at *p* > 0.05. F0, Control; F1, 2% *B. polymyxa*; F2, 2% *B. subtilis*; F3, 2% consortium of *B. subtilis* and *B. polymyxa*; F4, NPK synthetic.

### GC–MS profiling of secondary metabolites

**Figure 2:**
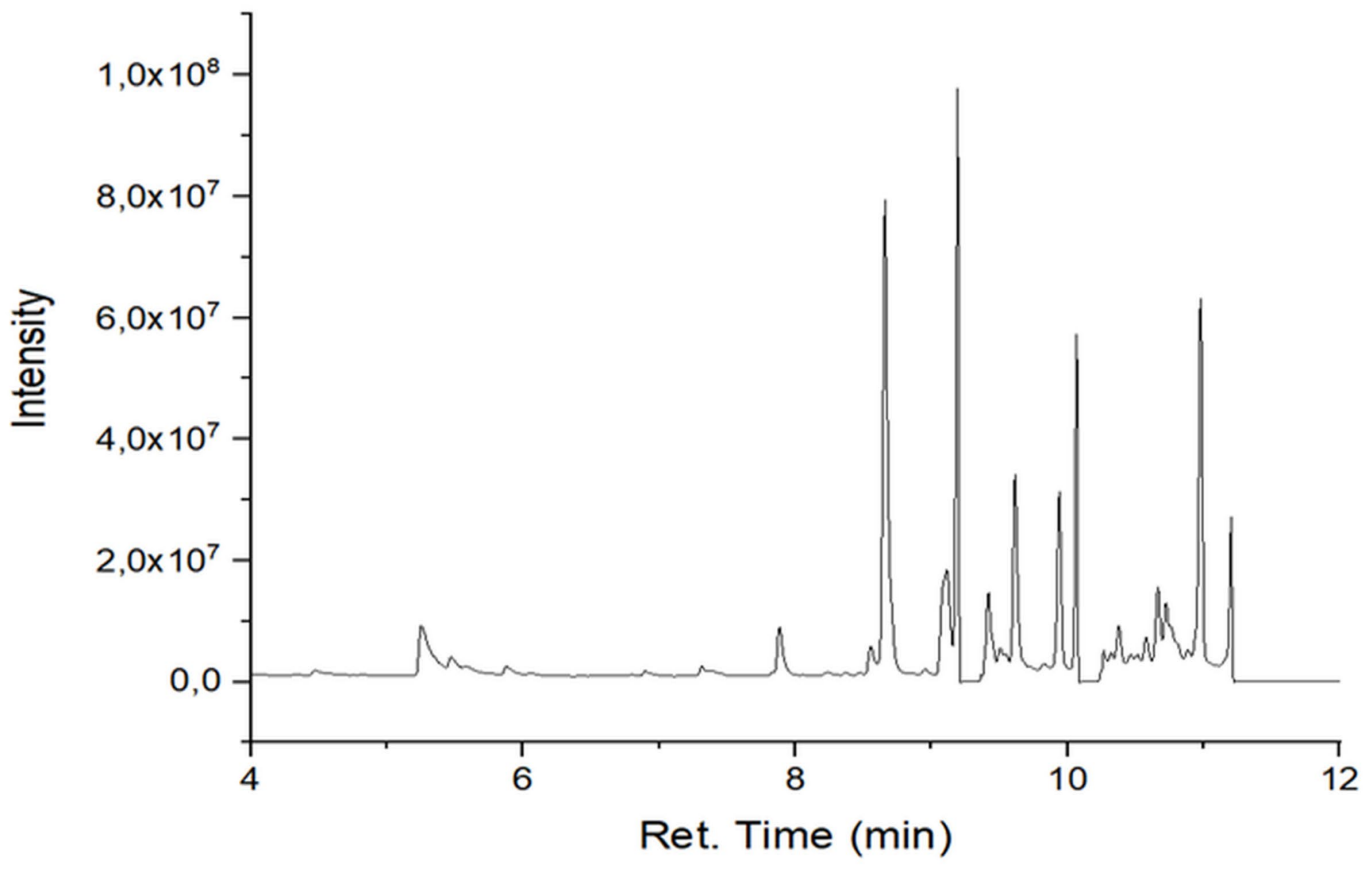
Chromatogram of extract of plants treated with F0.

**Figure 3:**
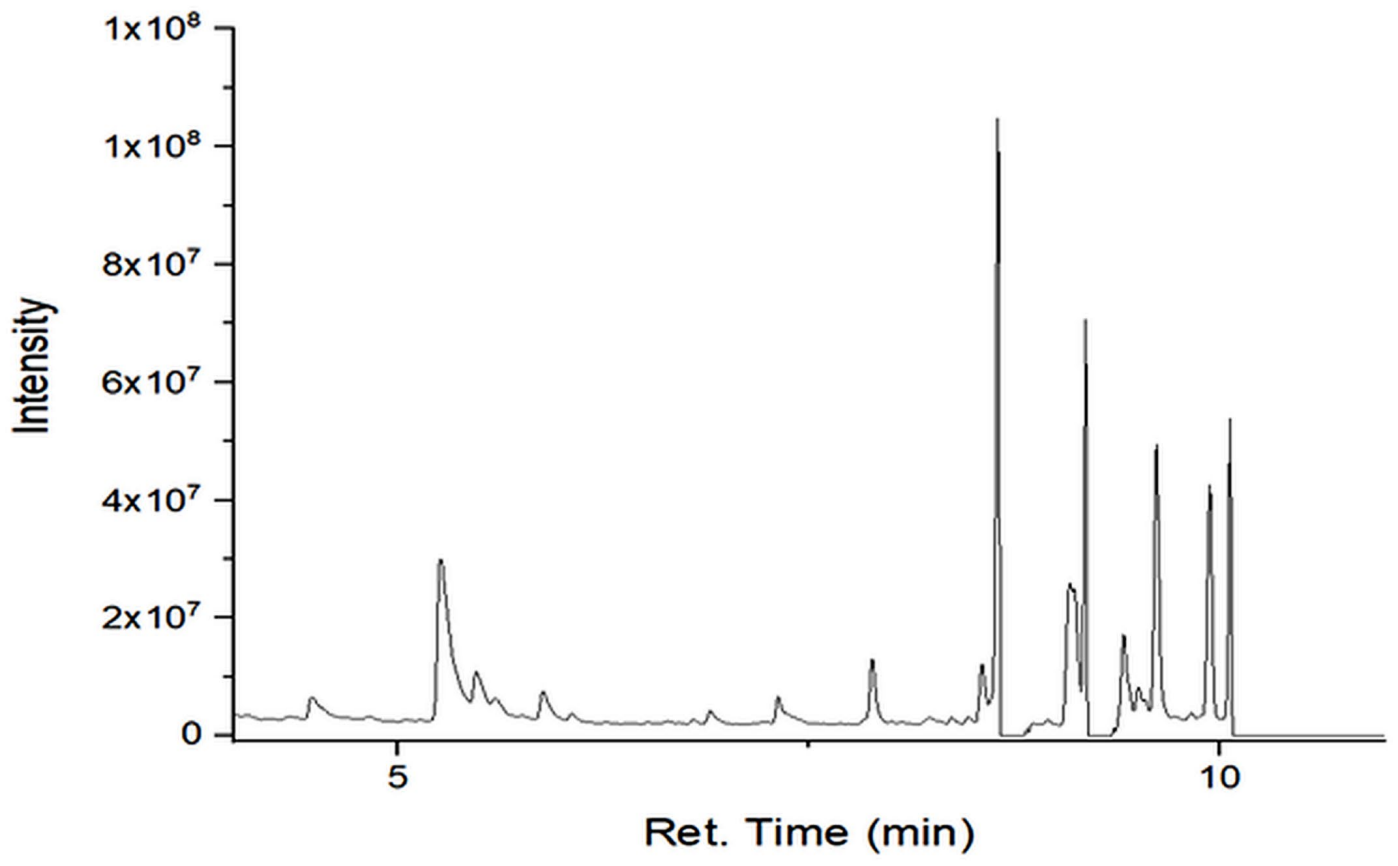
Chromatogram of extract of plant treated with F1.

**Figure 4:**
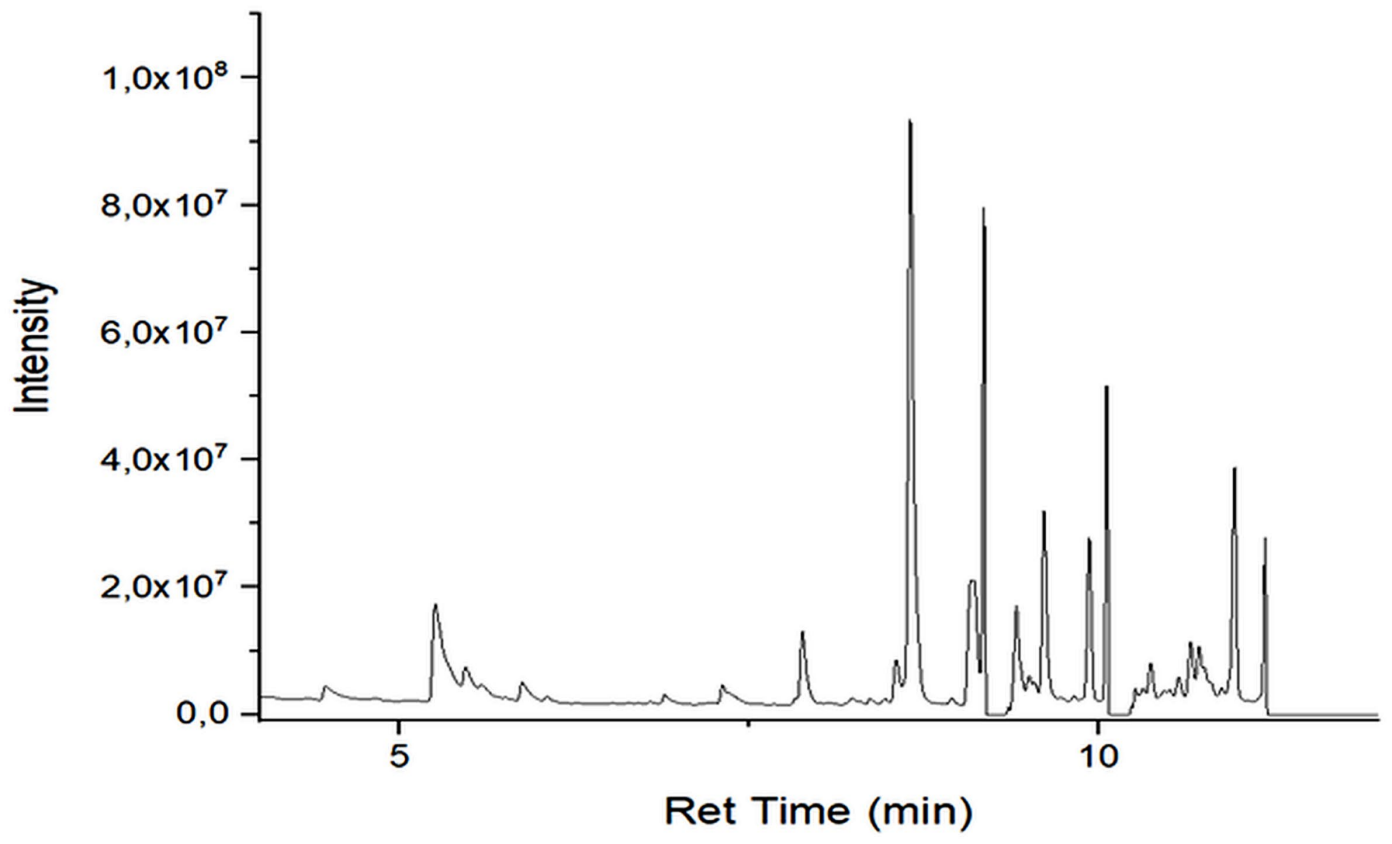
Chromatogram of extract of plant treated with F2.

**Figure 5:**
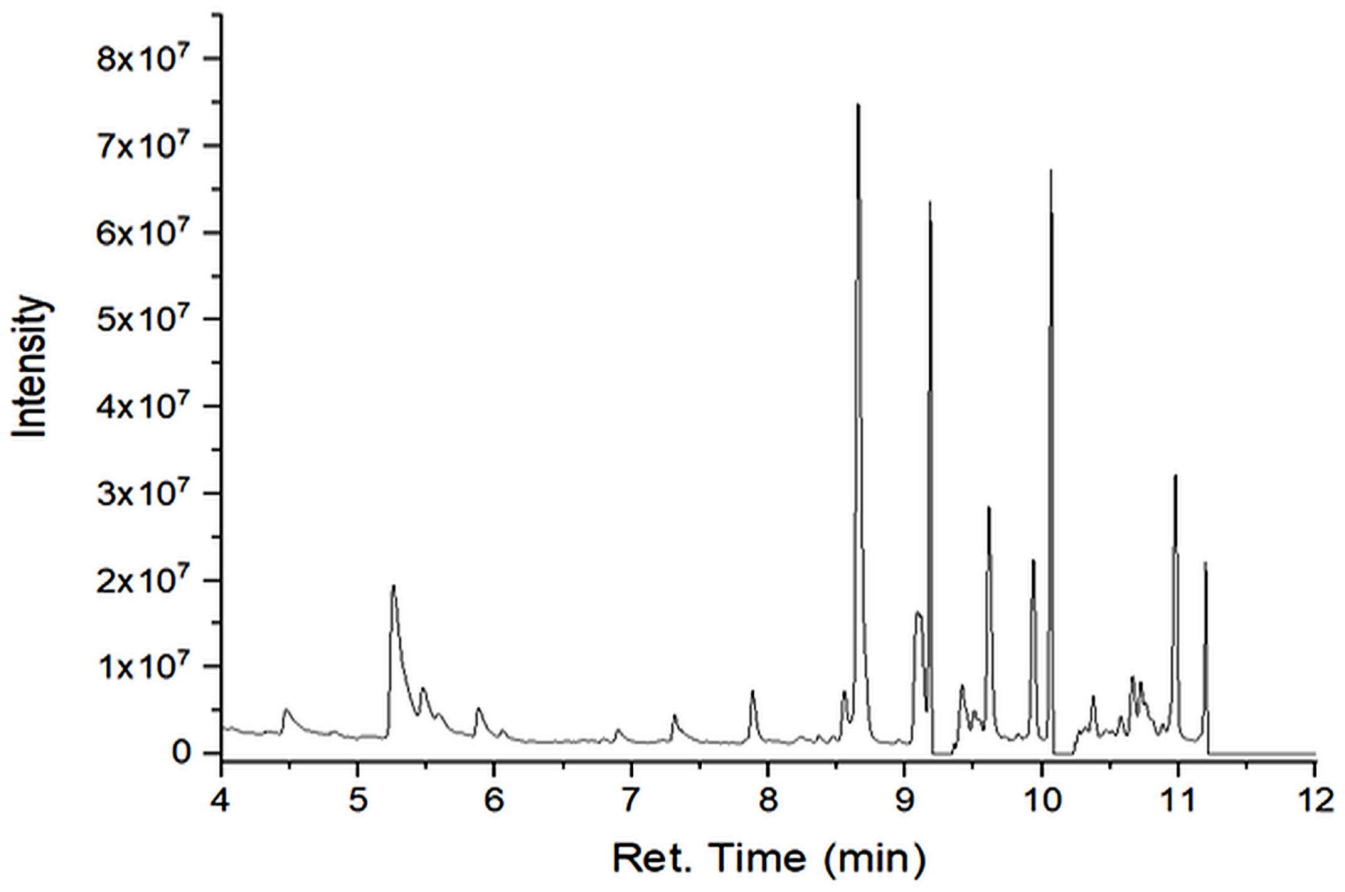
Chromatogram extract of plant treated with F3.

**Figure 6:**
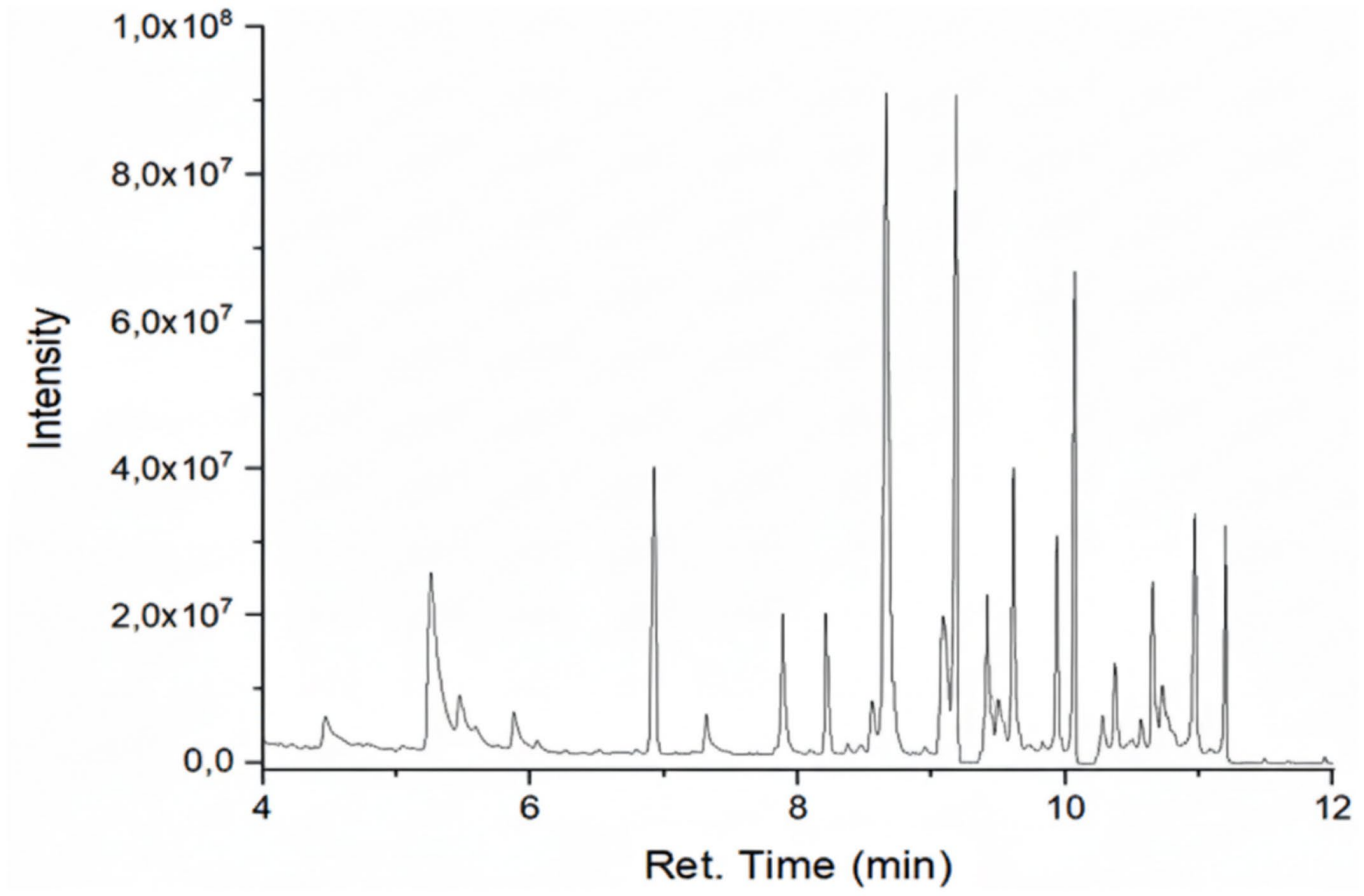
Chromatogram of extract of plants treated with F4.

GC–MS analysis revealed differences in the number, type, and relative abundance of metabolites among treatments (Table 5). The control treatment contained 19 compounds, including three sesquiterpenes with a total secondary metabolite area of 13.46%. Rhizobacterial treatments showed increased numbers and proportions of sesquiterpenes. The consortium treatment exhibited the highest total secondary metabolite area (67.47%). The NPK treatment showed a lower proportion of secondary metabolites compared with rhizobacterial treatments (Supplementary Table 5; Figures 2–6).

**Table 5.** Secondary metabolites and the area of GC-MS results.

Treatments	Types of compounds	Number of compounds	Area (%)	Class of compounds	Function
F0	Germacrene D, β-Caryophyllene, Elemene	3	13,46	Sesquiterpenes	Antifungal, Anticancer
F1	Germacrene D, β-Caryophyllene, Elemene, Valencene	4	34.98	Sesquiterpenes	Antimicrobial, Anticancer
F2	α-Humulene, Germacrene D, β-Caryophyllene, Elemene, Valencene	5	45.10	Sesquiterpenes	Antimicrobial, Anticancer
F3	α-Humulene, Germacrene D, β-Caryophyllene, Elemene, Valencene, Bicyclic sesquiterpene	6	67.47	Sesquiterpenes, terpenoid	Antimicrobial Anticancer
F4	Germacrene D, β-Caryophyllene, Elemene, Valencene	4	26.35	Sesquiterpenes	Antimicrobial, Anticancer

### Root colonization assessed by SEM

**Figure 7:**
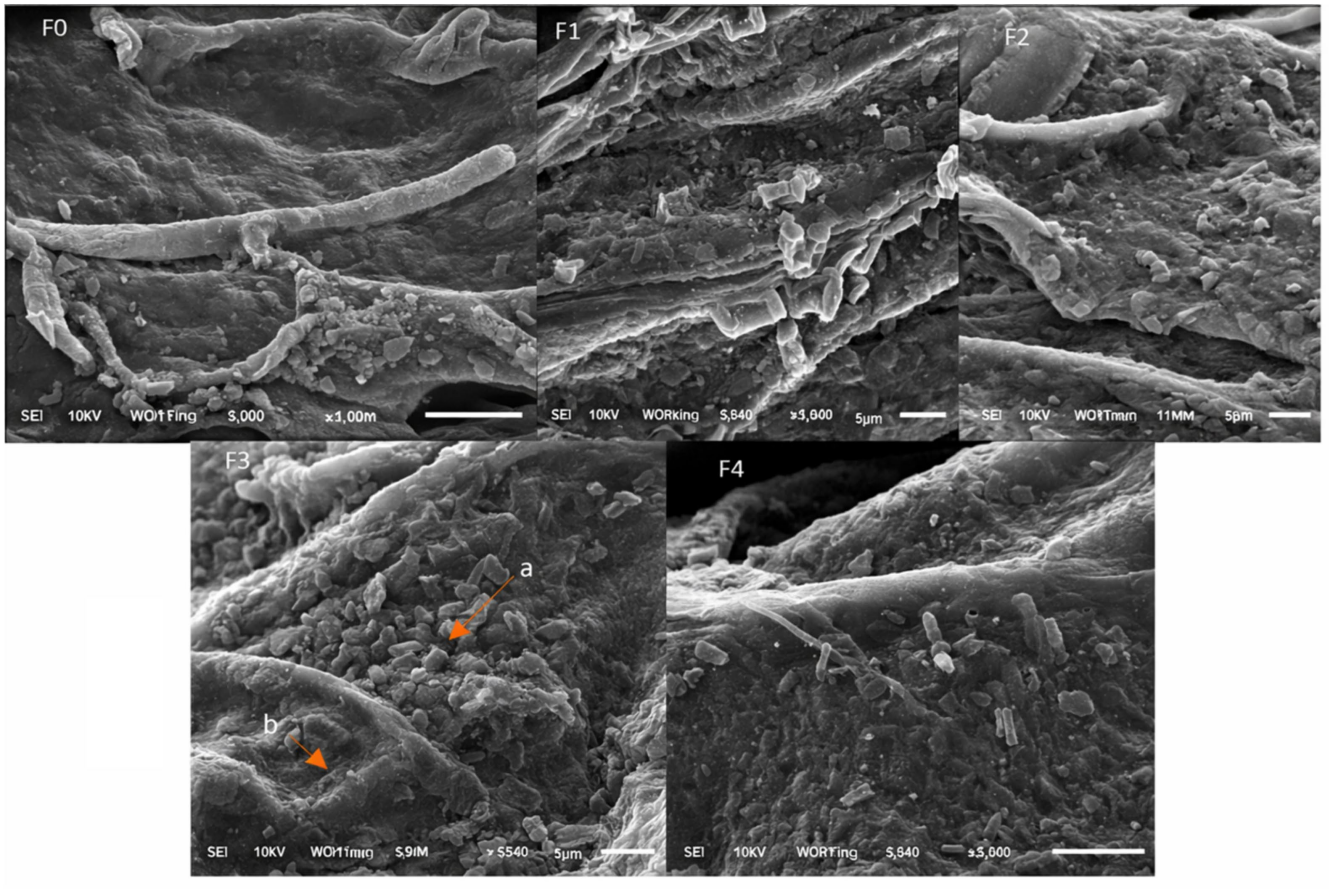
SEM colonization rhizobacteria (5,000x magnification). (F0) = Control, (F1) = *P. polymyxa*, (F2) = *B. subtilis*, consortium (F3), (F4) = NPK. a = Colonies bacteria, b = root of black turmeric plant.

SEM analysis demonstrated distinct differences in rhizobacterial colonization patterns (Figure 7). Minimal microbial presence was observed on the roots of control and NPK-treated plants. In contrast, dense and uniform colonization was observed in *P. polymyxa*-treated roots, localized colonization zones in *B. subtilis*-treated roots, and extensive overlapping colonization in consortium-treated roots.

## Discussion

Soil analysis after treatment demonstrated that nutrient availability was significantly influenced by fertilizer type and rhizobacterial inoculation. The highest soil N content was recorded in the NPK treatment, reflecting the immediate availability of synthetic nitrogen. In contrast, treatments inoculated with *Bacillus subtilis* exhibited the highest P and K contents, indicating the strong solubilization capacity of this bacterium. Compared with the control, all treatments showed improved soil nutrient status, with rhizobacteria-based treatments displaying particularly pronounced effects.

The enhanced P and K availability under *B. subtilis* treatment is consistent with its documented ability to solubilize insoluble phosphates and potassium-bearing minerals through organic acid production and enzymatic activity (Baba et al., 2021; Pradhan et al., 2025). Moreover, rhizobacteria-mediated nutrient mobilization is often more persistent than synthetic fertilizers due to effective root colonization and biofilm formation, which contribute to long-term nutrient cycling in the rhizosphere (Han et al., 2024). Although soil N levels under rhizobacterial treatments were lower than those under NPK fertilization, they were still significantly higher than the control, suggesting that biological N fixation provides a more gradual and environmentally sustainable nitrogen supply (Pathak et al., 2024). Sridhar et al. (2025a, 2025b) have reported an increase in soil nutrient content following the PGPR inoculation in PGPR consortia in the sesame rhizosphere. Jabborova et al. (2020, 2021) have also found an increase in soil nutrients and soil enzyme activity following the inoculation of PGPR in soybean and turmeric rhizosphere. These findings support the role of PGPR as a viable alternative to synthetic fertilizers for maintaining soil fertility while minimizing ecological risks.

Vegetative growth parameters of black turmeric, including plant height, leaf area, and root length, were significantly enhanced by rhizobacterial treatments compared with the control. The consortium treatment produced the highest overall plant growth, indicating synergistic interactions among bacterial members. Such synergism likely arises from combined production of phytohormones (IAA), enhanced nutrient mobilization, and stimulation of indigenous soil microbial communities (Niewiadomska et al., 2023).

Although the NPK treatment resulted in the largest leaf area, its effects are considered less sustainable for medicinal plants, as synthetic fertilizers can disrupt rhizosphere microbial balance. In contrast, rhizobacteria treatments significantly improved leaf development relative to the control, consistent with previous reports demonstrating that PGPR and cyanobacteria enhance leaf growth, anatomy, and physiological performance in various crops (Gashash et al., 2022; Yildiz et al., 2022).

Root length was most excellent under *B. subtilis* treatment, highlighting the strong root-colonizing ability of this bacterium. *B. subtilis* forms stable biofilms on root surfaces, improving nutrient acquisition and root architecture, thereby promoting overall plant vigor (Adjei et al., 2025). Chlorophyll content was highest in plants treated with *Paenibacillus polymyxa*, and all treatments significantly exceeded the control. Increased chlorophyll levels are likely linked to improved N and P availability and hormone production by rhizobacteria, which collectively enhance photosynthetic capacity. Similar effects of *B. subtilis* on chlorophyll accumulation have been reported under both normal and stress conditions (da Fonseca et al., 2022). Jabborova et al., 2025a,b have reported improvement in growth and physiological traits following the inoculation of rhizobacteria and mycorrhizae in black cumin and common beans.

Rhizome yield parameters revealed apparent differences among treatments. Although NPK fertilization produced the highest wet rhizome weight, it also resulted in the most significant weight loss during drying, reflecting high water content in tissues. This phenomenon is commonly associated with rapid vegetative growth driven by synthetic N inputs, which increase cell expansion and turgor pressure but do not necessarily enhance dry matter accumulation (Wang et al., 2024).

In contrast, *B. subtilis* treatment produced the highest dry rhizome weight, suggesting more efficient carbon assimilation and storage. This effect may be attributed to bacterial production of IAA and siderophores, which enhance nutrient uptake, photosynthesis, and assimilate partitioning toward storage organs (Backer et al., 2018). The slightly lower dry weight observed under consortium treatment may reflect a trade-off between growth and defense, where strong microbial signaling shifts plant metabolism toward defense-related pathways rather than biomass accumulation (Ahmed et al., 2024).

Wilt disease was observed exclusively in the NPK treatment, whereas no symptoms were detected in the control or rhizobacteria-treated plants. F3 contains the most secondary metabolite compounds such as *α*-Humulene, Germacrene D, *β*-Caryophyllene, Elemene, Valencene, Bicyclic sesquiterpene which have antimicrobial, antifungal and anticancer properties. Excessive nitrogen availability can increase tissue succulence, creating favorable conditions for soil-borne pathogens (Pei et al., 2025). Moreover, intensive NPK use disrupts beneficial microbial communities, thereby facilitating pathogen proliferation (Basu et al., 2021). The absence of disease under rhizobacterial treatments highlights the role of PGPR in suppressing pathogens through nutrient competition, antimicrobial compound production, and induction of systemic resistance (Al-Turki et al., 2023).

Sharma et al. (2025) have reported that amplified antifungal activity of *Trichoderma* sp., coupled with nanoparticles, improves antifungal activity against *Fusarium* wilt. Anbalagan et al. (2024) have found biocontrol potential of *Trichoderma asperellum* against *Fusarium nematode*. Dave et al. (2024) have reported the plant growth-promoting and wilt-controlling biopotential of actinomycetes and mycorrhizae. Sagar et al. (2024) have revealed the biopotential and biodiversity of *Bacillus* spp. Krishna et al. (2023) have also claimed anti-wilt activity of Triamcinolone acetonide-producing *Bacillus velezensis* YEBBR6 against *Fusarium oxysporum* f. sp. *Cubense.*

Phytochemical and antioxidant analyses revealed that rhizobacterial treatments significantly enhanced secondary metabolite accumulation compared with the control and NPK treatments. *P. polymyxa* treatment yielded the highest flavonoid and tannin contents, whereas *B. subtilis* treatment resulted in the highest phenolic, alkaloid, and antioxidant levels. Consortium treatments produced relatively stable but intermediate phytochemical levels, suggesting balanced microbial interactions (Parveen et al., 2026).

The increased accumulation of secondary metabolites under rhizobacterial treatments is consistent with previous findings in coffee, ginseng, and other medicinal plants (Suriani et al., 2024, 2025). PGPR-mediated improvements in N and P availability, combined with phytohormone production, stimulate key biosynthetic pathways such as the phenylpropanoid pathway, leading to enhanced synthesis of phenolics, flavonoids, tannins, and alkaloids (Backer et al., 2018; Faridvand et al., 2021).

Antioxidant activity showed an inverse relationship with IC₅₀ values, with *B. subtilis* treatment exhibiting the most substantial antioxidant potential, followed by *P. polymyxa* and consortium treatments. This enhancement is closely associated with elevated phenolic and flavonoid contents, which act as effective electron donors (Lasinskas et al., 2024; Martínez et al., 2022; Qi et al., 2025). Hamidian et al. (2023) have reported improved antioxidant responses in saffron inoculated with mycorrhiza. These findings further confirm that PGPR inoculation can modulate plant secondary metabolism toward higher medicinal value (Tang et al., 2024).

GC–MS profiling revealed distinct differences in the composition and abundance of metabolites among treatments. Rhizobacterial treatments, particularly the consortium, markedly increased both the diversity and relative abundance of sesquiterpene compounds compared with the control and NPK treatments. The consortium treatment exhibited the highest total area of secondary metabolites, indicating intense microbial stimulation of secondary metabolism (Parveen et al., 2026).

The enrichment of sesquiterpenes under bacterial treatments suggests complex microbe–plant signaling that activates specialized metabolic pathways. *P. polymyxa* and *B. subtilis* are known to produce enzymes, phytohormones, and elicitor molecules that enhance secondary metabolite biosynthesis (Sun et al., 2024). Conversely, the lower proportion of secondary metabolites under NPK treatment supports evidence that synthetic fertilizers favor primary metabolism while suppressing beneficial microbial interactions (Bai et al., 2020). Nithyapriya et al. (2024) in their study found improvement in nutrient content and plant growth in peanut due to inoculation with *Pseudomonas fluorescens*.

SEM observations confirmed that rhizobacterial treatments substantially enhanced root colonization compared with the control and NPK treatments. Minimal colonization in the control likely limited nutrient uptake efficiency and metabolite biosynthesis. *P. polymyxa* exhibited dense and uniform colonization, consistent with its biofilm-forming capacity and multifunctional plant growth-promoting traits (Timmusk et al., 2005; El-Saadony et al., 2024).

*B. subtilis* showed localized but strong adhesion to root surfaces, which is known to promote systemic resistance and nutrient mobilization, notably P and K (Santoyo et al., 2021). The consortium treatment displayed extensive overlapping colonization, reflecting high microbial density and signaling complexity. While this enhanced secondary metabolite production, it may have reduced nutrient uptake efficiency due to microbial competition, explaining the lower biomass but higher chemical diversity (Niewiadomska et al., 2023).

In contrast, NPK treatment resulted in minimal bacterial colonization, supporting evidence that synthetic fertilizers suppress beneficial rhizosphere microbes and destabilize root-associated biofilms (Jia et al., 2025). Reduced microbial interaction under NPK fertilization likely contributed to lower secondary metabolite production and increased disease susceptibility (Constantin et al., 2022).

## Conclusion

This study demonstrates that rhizobacterial inoculation exerts significant and consistent effects on the growth, yield, and metabolic quality of black turmeric compared with the untreated control. Inoculation with *P. polymyxa* and *B. subtilis* significantly enhanced plant growth, rhizome yield, and the accumulation of phytochemicals and antioxidants. Among the treatments, *B. subtilis* was the most effective in increasing rhizome dry weight, indicating its strong potential as a biofertilizer for improving yield. In contrast, both *P. polymyxa* and *B. subtilis* were particularly effective in enhancing the accumulation of major secondary metabolite groups, including tannins, alkaloids, phenolics, and flavonoids, as well as overall antioxidant activity.

GC–MS profiling further revealed that the bacterial consortium (*P. polymyxa* + *B. subtilis*) induced the greatest diversity and relative abundance of secondary metabolites, reflected by the highest total chromatographic area. In contrast, NPK fertilization predominantly promoted primary metabolite accumulation with comparatively limited stimulation of secondary metabolism. These findings indicate that microbial interactions play a central role in redirecting plant metabolic fluxes toward the biosynthesis of bioactive compounds.

The beneficial effects of rhizobacteria are attributable to multiple complementary mechanisms, including phytohormone (IAA) production, biological nitrogen fixation, phosphate solubilization, and effective root colonization, which collectively enhance nutrient availability, induce systemic resistance, and suppress wilt disease. Overall, rhizobacteria-based treatments offer a sustainable and environmentally friendly strategy to improve both yield and medicinal quality of black turmeric. Their application holds substantial promise for sustainable agriculture, particularly in the cultivation of herbal and food crops where high secondary metabolite content and reduced dependence on synthetic fertilizers are desirable.

## Data Availability

The original contributions presented in the study are included in the article/Supplementary material, further inquiries can be directed to the corresponding author.
