# Supplementary material for: PGPR-mediated enhancement of growth, phytochemical diversity, and metabolites in black turmeric (Curcuma caesia Roxb)
Source: Front Microbiol. 2026 Mar 13;17:1787961. doi: 10.3389/fmicb.2026.1787961 (PMC13021880; doi:10.3389/fmicb.2026.1787961)
Supplement: Supplementary file 1 [file Table_1.docx]

**Supplementary Table 1.** GC-MS analysis extract of plant treated with F0

| **Peak#** | **Retention Time** | **Area** | **Concentration (%)** | **Name of the compound** |
| --- | --- | --- | --- | --- |
| 1 | 5.253 | 26908803 | 2.75 | Bicyclo[2.2.1]heptan-2-one, 1,7,7-trimethyl-, (1S)- |
| 2 | 7.884 | 20625862 | 2.11 | Delta.-Elemene |
| 3 | 8.553 | 13716904 | 1.4 | BETA. ELEMENE |
| 4 | 8.659 | 2.04E+08 | 20.91 | BETA. ELEMENE |
| 5 | 9.112 | 77767139 | 7.96 | trans-Caryophyllene |
| 6 | 9.191 | 1.26E+08 | 12.86 | Germacrene B (CAS) 1,5-Cyclodecadiene, 1,5-dimethyl-8-(1-methylethylidene)-, (E,E)- |
| 7 | 9.418 | 38635391 | 3.95 | 1,2-dimethyl-2-vinyl-1-isopropenylcyclobutane |
| 8 | 9.5 | 16152394 | 1.65 | 1H-Cycloprop[e]azulene, 1a,2,3,4,4a,5,6,7b-octahydro-1,1,4,7-tetramethyl-, [1aR-(1a.alpha.,4.alpha.,4a.beta.,7b.alpha.)]- |
| 9 | 9.614 | 71892735 | 7.36 | Alpha.-Humulene |
| 10 | 9.939 | 57571380 | 5.89 | 1,6-Cyclodecadiene, 1-methyl-5-methylene-8-(1-methylethyl)-, [s-(E,E)]- |
| 11 | 10.066 | 67091932 | 6.87 | Benzofuran, 6-ethenyl-4,5,6,7-tetrahydro-3,6-dimethyl-5-isopropenyl-, trans- |
| 12 | 10.266 | 9365924 | 0.96 | ND |
| 13 | 10.377 | 25586789 | 2.62 | 4,4-Dimethyl-3-(3-methyl-3-buten-1-yliden)-2-methylidenbicyclo[4.1.0]heptane |
| 14 | 10.576 | 8390363 | 0.86 | Alpha.-Gurjunene |
| 15 | 10.664 | 28091191 | 2.87 | bicyclogermacrene |
| 16 | 10.723 | 23363711 | 2.39 | NEOISOLONGIFOLEN, 8,9-DEHYDRO- |
| 17 | 10.765 | 14216122 | 1.45 | 2-Cyclohexen-1-one, 2-methyl-5-(1-methylethenyl)- (CAS) 2-Methyl-5-isopropenyl-2-cyclohexenone |
| 18 | 10.978 | 1.16E+08 | 11.91 | Germacrene B (CAS) 1,5-Cyclodecadiene, 1,5-dimethyl-8-(1-methylethylidene)-, (E,E)- |
| 19 | 11.2 | 31332908 | 3.21 | PENTAN-1,3-DIOLDIISOBUTYRATE, 2,2,4-TRIMETHYL- |

**Supplementary Table 2.** GC-MS analysis extract of plant treated with F1

| **Peak** | **Retention Time** | **Area** | **Concentration (%)** | **Name of the compound** |
| --- | --- | --- | --- | --- |
| 1 | 4.48 | 16910138 | 1.69 | Dodecane (CAS) n-Dodecane |
| 2 | 5.262 | 1.57E+08 | 15.66 | Bicyclo [2.2.1]heptan-2-one, 1,7,7-trimethyl-, (1S)- |
| 3 | 5.479 | 35483003 | 3.54 | Borneol |
| 4 | 5.593 | 12393413 | 1.24 | ND |
| 5 | 5.884 | 18810874 | 1.88 | Dodecane (CAS) n-Dodecane |
| 6 | 6.902 | 6484520 | 0.65 | Dodecane, 2,6,11-trimethyl- |
| 7 | 7.315 | 7687420 | 0.77 | Tridecane (CAS) n-Tridecane |
| 8 | 7.886 | 27794913 | 2.77 | Delta.-Elemene |
| 9 | 8.465 | 6828216 | 0.68 | Trichloroacetic acid, dodec-9-ynyl ester |
| 10 | 8.553 | 32908066 | 3.29 | BETA. ELEMENE |
| 11 | 8.65 | 1.46E+08 | 14.55 | BETA. ELEMENE |
| 12 | 9.087 | 63795374 | 6.37 | Cyclohexane, 1-ethenyl-1-methyl-2-(1-methylethenyl)-4-(1-methylethylidene)- |
| 13 | 9.115 | 49462816 | 4.94 | trans-Caryophyllene |
| 14 | 9.182 | 80650829 | 8.05 | Germacrene B (CAS) 1,5-Cyclodecadiene, 1,5-dimethyl-8-(1-methylethylidene)-, (E,E)- |
| 15 | 9.415 | 51089699 | 5.1 | 1,2-dimethyl-2-vinyl-1-isopropenylcyclobutane |
| 16 | 9.505 | 17576109 | 1.75 | Alpha.-Gurjunene |
| 17 | 9.55 | 9572087 | 0.96 | GERMACRENE-D |
| 18 | 9.614 | 1.02E+08 | 10.22 | Alpha.-Humulene |
| 19 | 9.939 | 94061400 | 9.39 | 1,6-Cyclodecadiene, 1-methyl-5-methylene-8-(1-methylethyl)-, [s-(E,E)]- |
| 20 | 10.062 | 65299869 | 6.52 | Curzerene |

**Supplementary Table 3**. GC-MS analysis extract of plant treated with F2

| **Peak#** | **Retention Time** | **Area** | **Concentration (%)** | **Name of compounds** |
| --- | --- | --- | --- | --- |
| 1 | 5.258 | 84939276 | 7.71 | Camphor |
| 2 | 5.474 | 13168417 | 1.19 | Bicyclo [2.2.1]heptan-2-ol, 1,7,7-trimethyl-, exo- (CAS) Isoborneol |
| 3 | 5.882 | 11733526 | 1.06 | Dodecane (CAS) n-Dodecane |
| 4 | 7.886 | 33077382 | 3 | Delta.-Elemene |
| 5 | 8.553 | 19106286 | 1.73 | BETA. ELEMENE |
| 6 | 8.656 | 2.58E+08 | 23.39 | BETA. ELEMENE |
| 7 | 9.11 | 95356917 | 8.65 |  |
| 8 | 9.182 | 89998635 | 8.16 | Germacrene B (CAS) 1,5-Cyclodecadiene, 1,5-dimethyl-8-(1-methylethylidene)-, (E,E)- |
| 9 | 9.413 | 49805717 | 4.52 | 1,5-CYCLOOCTADIENE, 3,4-DIMETHYL- |
| 10 | 9.505 | 13334863 | 1.21 | Seychellene |
| 11 | 9.545 | 7840586 | 0.71 | GERMACRENE-D |
| 12 | 9.612 | 70815420 | 6.42 | Alpha.-Humulene |
| 13 | 9.936 | 51560640 | 4.68 | 1,6-Cyclodecadiene, 1-methyl-5-methylene-8-(1-methylethyl)-, [s-(E,E)]- |
| 14 | 10.062 | 58342132 | 5.29 | Benzofuran, 6-ethenyl-4,5,6,7-tetrahydro-3,6-dimethyl-5-isopropenyl-, trans- |
| 15 | 10.318 | 9984059 | 0.91 | 1(10),4-aromedenedradiene |
| 16 | 10.374 | 22942306 | 2.08 | 4,4-Dimethyl-3-(3-methyl-3-buten-1-yliden)-2-methylidenbicyclo[4.1.0]heptane |
| 17 | 10.513 | 18406596 | 1.67 | Alpha.-Santalol |
| 18 | 10.574 | 15564609 | 1.41 | Alpha.-Gurjunene |
| 19 | 10.662 | 27970184 | 2.54 | Bicyclogermacrene |
| 20 | 10.72 | 21685940 | 1.97 | Neoisolongifolene, 8,9-dehydro- |
| 21 | 10.76 | 14336038 | 1.3 | 2-Cyclohexen-1-one, 2-methyl-5-(1-methylethenyl)- (CAS) 2-Methyl-5-isopropenyl-2-cyclohexenone |
| 22 | 10.81 | 7587420 | 0.69 | Thujopsene-I3 |
| 23 | 10.973 | 76959694 | 6.98 | Germacrene B (CAS) 1,5-Cyclodecadiene, 1,5-dimethyl-8-(1-methylethylidene)-, (E,E)- |
| 24 | 11.192 | 29974075 | 2.72 | 2,2,4-Trimethyl-1,3-pentanediol diisobutyrate |

**Supplementary Table 4.** GC-MS analysis of extract of plant treated with F3

| **Peak#** | **Retention Time** | **Area** | **Concentration (%)** | **Name of the compound** |
| --- | --- | --- | --- | --- |
| 1 | 5.257 | 1E+08 | 11.67 | Camphor |
| 2 | 5.476 | 15496811 | 1.8 | Bicyclo[2.2.1]heptan-2-ol, 1,7,7-trimethyl-, exo- (CAS) Isoborneol |
| 3 | 7.316 | 5016300 | 0.58 | Hexadecane (CAS) n-Hexadecane |
| 4 | 7.885 | 14853407 | 1.73 | Delta.-Elemene |
| 5 | 8.554 | 15594278 | 1.81 | BETA. ELEMENE |
| 6 | 8.657 | 2E+08 | 23.23 | Cyclohexane, 1-ethenyl-1-methyl-2,4-bis(1-methylethenyl)-, [1S-(1.alpha.,2.beta.,4.beta.)]- |
| 7 | 9.088 | 71622487 | 8.33 | Valencene |
| 8 | 9.182 | 71950531 | 8.37 | Germacrene B (CAS) 1,5-Cyclodecadiene, 1,5-dimethyl-8-(1-methylethylidene)-, (E,E)- |
| 9 | 9.414 | 27149600 | 3.16 | 1,2-dimethyl-2-vinyl-1-isopropenylcyclobutane |
| 10 | 9.505 | 11003273 | 1.28 | 1H-Cycloprop[e]azulene, 1a,2,3,4,4a,5,6,7b-octahydro-1,1,4,7-tetramethyl-, [1aR-(1a.alpha.,4.alpha.,4a.beta.,7b.alpha.)]- |
| 11 | 9.55 | 5832422 | 0.68 | GERMACRENE-D |
| 12 | 9.612 | 63005638 | 7.33 | .alpha.-Humulene |
| 13 | 9.937 | 40958886 | 4.77 | 1,6-Cyclodecadiene, 1-methyl-5-methylene-8-(1-methylethyl)-, [s-(E,E)]- |
| 14 | 10.066 | 79452258 | 9.25 | Curzerene |
| 15 | 10.374 | 11347985 | 1.32 | 4,4-Dimethyl-3-(3-methylbut-3-enylidene)-2-methylenebicyclo[4.1.0]heptane |
| 16 | 10.661 | 17172999 | 2 | bicyclogermacrene |
| 17 | 10.721 | 13802662 | 1.61 | NEOISOLONGIFOLEN, 8,9-DEHYDRO- |
| 18 | 10.76 | 8851995 | 1.03 | 2-Cyclohexen-1-one, 2-methyl-5-(1-methylethenyl)- (CAS) 2-Methyl-5-isopropenyl-2-cyclohexenone |
| 19 | 10.973 | 61908870 | 7.2 | Germacrene B (CAS) 1,5-Cyclodecadiene, 1,5-dimethyl-8-(1-methylethylidene)-, (E,E)- |
| 20 | 11.196 | 24468457 | 2.85 | PENTAN-1,3-DIOLDIISOBUTYRATE, 2,2,4-TRIMETHYL- |

**Supplementary Table 5**. GC-MS analysis of extract of plant treated with F4

| **Peak No** | **Retention Time** | **Area** | **Concen-**  **-tration (%)** | **Name of the compound** |
| --- | --- | --- | --- | --- |
| 1 | 5.26 | 106798956 | 10.57 | Bicyclo [2.2.1]heptan-2-one, 1,7,7-trimethyl-, (1S)- |
| 2 | 5.476 | 15914492 | 1.57 | Bicyclo[ 2.2.1]heptan-2-ol, 1,7,7-trimethyl-, exo- (CAS) Isoborneol |
| 3 | 7.317 | 4793040 | 0.47 | Tridecane |
| 4 | 7.886 | 27389193 | 2.71 | Delta.-Elemene |
| 5 | 8.556 | 17343103 | 1.72 | BETA. ELEMENE |
| 6 | 8.658 | 246067217 | 24.34 | BETA. ELEMENE |
| 7 | 9.091 | 79719645 | 7.89 | Valencene |
| 8 | 9.186 | 101982940 | 10.09 | Germacrene B (CAS) 1,5-Cyclodecadiene, 1,5-dimethyl-8-(1-methylethylidene)-, (E,E)- |
| 9 | 9.36 | 1338608 | 0.13 | HEXABORANE-12 |
| 10 | 9.415 | 38290098 | 3.79 | 1,2-dimethyl-2-vinyl-1-isopropenylcyclobutane |
| 11 | 9.502 | 15243600 | 1.51 | 1H-Cycloprop[e]azulene, decahydro-1,1,7-trimethyl-4-methylene-, [1aR-(1a.alpha.,4a.beta.,7.alpha.,7a.beta.,7b.alpha.)]- |
| 12 | 9.555 | 6292100 | 0.62 | GERMACRENE-D |
| 13 | 9.614 | 69360727 | 6.86 | Alpha.-Humulene |
| 14 | 9.938 | 43796401 | 4.33 | 1,6-Cyclodecadiene, 1-methyl-5-methylene-8-(1-methylethyl)-, [s-(E,E)]- |
| 15 | 10.066 | 70266652 | 6.95 | Curzerene |
| 16 | 10.24 | 1615076 | 0.16 | HEXABORANE-12 |
| 17 | 10.265 | 9949639 | 0.98 | 1H-Cycloprop[e]azulen-4-ol, decahydro-1,1,4,7-tetramethyl-, [1ar-(1a.alpha.,4.beta.,4a.beta.,7.alpha.,7a.beta.,7b.alpha.)]- |
| 18 | 10.374 | 12468211 | 1.23 | 4,4-Dimethyl-3-(3-methylbut-3-enylidene)-2-methylenebicyclo[4.1.0]heptane |
| 19 | 10.574 | 5458049 | 0.54 | 1H-Cycloprop[e]azulene, 1a,2,3,4,4a,5,6,7b-octahydro-1,1,4,7-tetramethyl-, [1aR-(1a.alpha.,4.alpha.,4a.beta.,7b.alpha.)]- |
| 20 | 10.664 | 17174236 | 1.7 | Germacrene B (CAS) 1,5-Cyclodecadiene, 1,5-dimethyl-8-(1-methylethylidene)-, (E,E)- |
| 21 | 10.722 | 14912296 | 1.48 | NEOISOLONGIFOLEN, 8,9-DEHYDRO- |
| 22 | 10.76 | 9625864 | 0.95 | 1H-2-Benzopyran, 3,5,8,8a-tetrahydro-6-methyl- |
| 23 | 10.82 | 4433518 | 0.44 | 2,4A,8,8-TETRAMETHYL-1,1A,4,4A,5,6,7,8-OCTAHYDRO-CYCLOPROPA[D]NAPHTHALENE |
| 24 | 10.974 | 66226784 | 6.55 | Germacrene B (CAS) 1,5-Cyclodecadiene, 1,5-dimethyl-8-(1-methylethylidene)-, (E,E)- |
| 25 | 11.196 | 24392244 | 2.41 | PENTAN-1,3-DIOLDIISOBUTYRATE, 2,2,4-TRIMETHYL- |
